# ‘Peace’ and ‘life worthwhile’ as measures of spiritual well-being in African palliative care: a mixed-methods study

**DOI:** 10.1186/1477-7525-11-94

**Published:** 2013-06-10

**Authors:** Lucy Selman, Peter Speck, Marjolein Gysels, Godfrey Agupio, Natalya Dinat, Julia Downing, Liz Gwyther, Thandi Mashao, Keletso Mmoledi, Tony Moll, Lydia Mpanga Sebuyira, Barbara Ikin, Irene J Higginson, Richard Harding

**Affiliations:** 1 King’s College London, Department Palliative Care, Policy and Rehabilitation, Cicely Saunders Institute, Bessemer Road, Denmark Hill, London SE5 9PJ, UK; 2 Centre for Social Science and Global Health, University of Amsterdam, Oudezijds Achterburgwal 185, Amsterdam 1012, The Netherlands; 3 Hospice Africa Uganda, Plot 130 Makindye Road, PO Box, Kampala 7757, Uganda; 4 Division of Palliative Care, The University of the Witwatersrand, PO Box 212, Pimville, Soweto 1808, Johannesburg, Gauteng, South Africa; 5 African Palliative Care Association, PO Box 72518, Kampala, Uganda; 6 Hospice Palliative Care Association of South Africa, PO Box 38785 Howard Place, 7450Suite 11a, Lonsdale Building, Lonsdale Way, Pinelands, Cape Town, Western Cape 7430, South Africa; 7 Palliative Medicine Unit, University of Cape Town, Anzio Road, Observatory, Cape Town, Western Cape 7925, South Africa; 8 Church of Scotland Hospital, P/Bag X502, Tugela Ferry, KwaZulu Natal 3010, South Africa; 9 South Coast Hospice, P.O. Box 504, Port Shepstone, KwaZulu Natal 4240, South Africa

**Keywords:** Outcome measurement, Spiritual well-being, Content validity, Construct validity, Sub-Saharan Africa, Palliative care

## Abstract

**Background:**

Patients with incurable, progressive disease receiving palliative care in sub-Saharan Africa experience high levels of spiritual distress with a detrimental impact on their quality of life. Locally validated measurement tools are needed to identify patients’ spiritual needs and evaluate and improve spiritual care, but up to now such tools have been lacking in Africa. The African Palliative Care Association (APCA) African Palliative Outcome Scale (POS) contains two items relating to *peace* and *life worthwhile*. We aimed to determine the content and construct validity of these items as measures of spiritual wellbeing in African palliative care populations.

**Methods:**

The study was conducted at five palliative care services, four in South Africa and one in Uganda. The mixed-methods study design involved: (1) cognitive interviews with 72 patients, analysed thematically to explore the items’ content validity, and (2) quantitative data collection (n = 285 patients) using the POS and the Spirit 8 to assess construct validity.

**Results:**

(1) *Peace* was interpreted according to the themes ‘perception of self and world’, ‘relationship to others’, ‘spiritual beliefs’ and ‘health and healthcare’. *Life worthwhile* was interpreted in relation to ‘perception of self and world’, ‘relationship to others’ and ‘identity’. (2) Conceptual convergence and divergence were also evident in the quantitative data: there was moderate correlation between *peace* and Spirit 8 *spiritual well*-*being* (r = 0.46), but little correlation between *life worthwhile* and Spirit 8 *spiritual well*-*being* (r = 0.18) (both p < 0.001). Correlations with Spirit 8 items were weak to moderate.

**Conclusions:**

Findings demonstrate the utility of POS items *peace* and *life worthwhile* as distinct but related measures of spiritual well-being in African palliative care. *Peace* and *life worthwhile* are brief and simple enough to be integrated into routine practice and can be used to measure this important but neglected outcome in this population.

## Background

The detrimental impact of progressive and life-limiting diseases such as HIV and advanced cancer on spiritual aspects of well-being is well evidenced [1]– [3], including in sub-Saharan Africa [4,5]. Spiritual care, understood to include care relating to existential concerns (e.g. meaning and purpose) as well as religious guidance and ritual, is therefore an essential component of the holistic, person-centred palliative care approach [6].

Yet despite evidence of spiritual needs and the stipulations of international policy guidance [7]– [9], patients’ spiritual needs are poorly assessed and met [10]– [12]. There is evidence from North America that neglect of spiritual needs is associated with reduced quality of life and satisfaction with care [13,14] and increased healthcare costs at the end of life [15]. In a study of 285 patients with HIV or advanced cancer in South Africa and Uganda, 21-58% scored poorly on spiritual well-being [16], and patients weighted feeling at peace and having a sense of meaning in life more highly than physical comfort [5], suggesting that the neglect of spiritual needs is likely to have a particularly detrimental effect on quality of life in this population.

One of the reasons why spiritual needs are poorly managed in clinical practice is a lack of confidence and competence among healthcare staff in identifying, assessing and discussing spiritual distress [17]. Formal tools for assessment and measurement are needed to ensure that spiritual care is equitable, spiritual needs are identified and quality of care is evaluated [18]– [21].

Owing to the cultural variability in conceptualisations of spiritual well-being [22,23] and the specific concerns that arise in incurable, progressive disease, it is essential that measurement tools for use in sub-Saharan palliative care are locally validated in clinically relevant populations [24,25]. The African Palliative Care Association (APCA) African Palliative Outcome Scale (POS) [26,27] is a patient-reported outcome measure developed and validated in palliative care populations in eight African countries. Two items in the tool assess psycho-spiritual outcomes: ‘Over the past three days, have you felt at peace?’ and ‘Over the past three days, have you felt that life is worthwhile?’. These items (*peace* and *life worthwhile*) were developed through expert consultation and piloting with staff and patients, have good face validity as judged by experts in the field, are well comprehended by patients and show good test-retest reliability and sensitivity to change [26]. Recent factor analysis of the tool found the two items load on an existential well-being factor, suggesting these items form a unidimensional measure which might be used to measure spiritual well-being and screen for spiritual distress [28].

However, although the APCA African POS is established as a valid measure of palliative outcomes in sub-Saharan Africa, validity is dependent on use and is a question of degree. The content and construct validity of the *peace* and *life worthwhile* items have not previously been explored in an in depth manner, and this is crucial if the items are to be used to measure spiritual well-being and screen for spiritual distress in this population.

We therefore aimed to explore the constructs measured by the *peace* and *life worthwhile* items and how they relate to each other, to determine the items’ utility in this setting. The study objectives were to (1) investigate the content validity of the items through exploring how they are interpreted by patients and (2) investigate the items’ construct validity by examining their relationship with indicators of spiritual well-being from the Spirit 8, an existing measure of spiritual well-being validated in this population [16].

## Method

### Study design

A mixed-methods study design was used to meet the study objectives: (1) cognitive interviews with patients exploring their interpretations of ‘feeling at peace’ and ‘feeling life is worthwhile’; (2) patient survey, using the POS and the Spirit 8. Cognitive interviewing is an established technique to ascertain content validity, including in advanced disease populations [29].

### Setting

Three palliative care facilities in South Africa and one in Uganda participated in the cognitive interviews and survey. An additional palliative care service in South Africa took part in the survey only. Inclusion criteria were: established palliative care services able to support research and fulfil recruitment criteria for the study. Services were selected to represent a diverse range of service types (home-based care and inpatient units) and locations (rural, urban township and urban) to enhance generalisability of findings [30].

All services aimed to provide holistic palliative care in line with the WHO definition [6], provided by multi-professional teams that included doctors, nurses, caregivers/nursing assistants, social workers, access to spiritual care and (at two sites) counsellors.

### Recruitment and sampling

At each palliative care site, a trained, locally-based palliative care researcher approached eligible patients.

For cognitive interviewing, a purposive sample was recruited over 10 weeks. Recruitment of 15–20 patients at each site was estimated to achieve data saturation when data from each site were analysed separately [31], giving a target sample of 60–80 patients overall. The purposive sampling frame addressed diagnosis, place of care (community/inpatient/outpatient), gender, location and ethnic group, in order to generate a maximum variation sample. For quantitative data collection, patients were recruited consecutively.

Study inclusion criteria were: adult (at least 18 years’ old) patient, able to give informed consent, judged physically and mentally well enough to participate by their clinical staff, and able to speak either English or one of six local languages fluently (isiXhosa, isiZulu, SeSotho, SeTswana, Luganda and Runyoro).

The study was reviewed and approved by the Ethical Review Boards of the Universities of Cape Town, KwaZulu Natal and Witwatersrand, the Ugandan National Council for Science and Technology, Hospice Africa Uganda, and the Hospice Palliative Care Association of South Africa. Informed written consent was gained prior to the interviews, with illiterate participants being read the information sheet and consent form and marking their consent [32].

### Data collection

#### Cognitive interviews

The POS *peace* and *life worthwhile* items were read to participants, who were asked if they understood the question, what they thought the question meant, and how they would decide on an answer. The researchers at each site recorded, transcribed and translated the interviews, discussing the translation of complex terms or phrases with facility staff members and the Principal Investigators (PIs) at each site, who reviewed the transcripts to check the accuracy of translations. Anonymised transcripts were sent to the first author (LS) for analysis.

#### Quantitative data

Researchers completed the Spirit 8 and the POS with each participant. The Spirit 8 [16] is an eight-item scale developed from the Missoula Vitas Quality of Life Index (MVQOLI), a tool developed in the USA [33] and validated in Uganda [34]. All items refer to how the patient currently feels, and are scored on a 1 - 5 scale ( 5 indicating a better outcome). Seven of the eight items require choosing between positive and negative statements. A *spiritual wellbeing* score is produced by summing the scores for all items (possible score 8–40). Factor and Rasch analyses support the use of the Spirit 8 as a unidimensional measure of spiritual well-being (α = 0.73) in this population [16].

The POS is a 10-item self-reported questionnaire that assesses 7 patient outcomes (including *peace* and *life worthwhile*) and 3 carer outcomes [26,27]. Items ask about the previous 3 days, and responses are given on a six-point scale (0–5). POS items can be considered individually to highlight and track specific areas of potential need or according to three factors (physical and psychological wellbeing, interpersonal wellbeing and existential wellbeing) [28].

#### Demographic data

Demographic data were: age, gender, location of home (urban/peri-urban/rural), household size, whether or not the patient had children (and how many), primary place of palliative care, weeks under palliative care, primary diagnosis and functional status. For HIV + patients, data were collected on receipt of antiretroviral therapy (ART), AIDS diagnosis, and latest CD4 count. A diagnosis of AIDS was defined as the presence of a CD4 cell count < 200 cells/μl or an AIDS-defining illness such as extrapulmonary tuberculosis. The Eastern Cooperative Oncology Group (ECOG) performance status was used to measure functional status from 0 (no disability) to 4 (completely disabled) [35]. Spiritual belief system was elicited during cognitive interviews.

#### Translation

Data collection tools, information sheets and informed consent forms were translated from English into six common local languages (see *Inclusion criteria*). The University of KwaZulu Natal performed the isiZulu translation and the University of Cape Town the isiXhosa translation. Other translations were conducted by the PI and researcher at the participating facilities and cross-checked by bilingual members of staff.

### Analysis

#### Cognitive interview data

Thematic content analysis [36,37] of the transcripts was conducted in NVivo v9 to identify interpretations of *peace* and *life worthwhile*. Construction of the coding frame followed best practice [38,39]: two researchers (LS & PS) independently constructed coding frames based on a purposive sample of five transcripts from each site including patients with different demographic characteristics (age, gender, location of home). A constant comparison, iterative approach was adopted to create themes and sub-themes [39]. Generated themes were sorted by levels of generality and grouped under a smaller number of broader, higher-order themes within a conceptually clear framework that facilitated deeper analysis and thematic integration. LS and PS compared coding frames, discussed differences and agreed on a final coding frame for the analysis. Coding was conducted by LS, with a random sample of 12 transcripts double coded (PS) to compare inter-rater coding. Discrepancies in coding were resolved through discussion. Preliminary findings were discussed with the rest of the research team to increase validity.

In reporting, all qualitative data are anonymised. Identification codes represent the site (A-D) and participant code.

#### Quantitative data

We tested for correlations between the Spirit 8 and the POS *peace* and *life worthwhile* items using Spearman’s Rank tests in SPSS v19. Hypotheses for testing were generated on the basis of findings from a content analysis of the concepts occurring in measures of spirituality in palliative care identified through systematic review [24]. The content analysis categorised feeling at peace as an indicator of spiritual well-being together with other concepts such as hope and regret that relate to coming to terms with illness and approaching death. Steinhauser et al. also report that among patients with incurable illness, attention to issues of peacefulness was related to an antecedent, broader theme of “completion” or life closure [40]. We therefore hypothesised that *peace* would correlate most highly with Spirit 8 items related to preparation for death and to feeling at peace, i.e. (i) Q1 (‘My affairs are in order; I could die today with a clear mind’), (ii) Q2 (‘I feel generally at peace and prepared to leave this life’) and (iii) Q7 (‘As the end of my life approaches, I am comfortable with the thought of my own death’).

In the content analysis the concept of ‘life worth’ occurred under the broader theme of items related to a patient’s outlook on life or self, which also included the concept of viewing life as meaningful/meaningless and as gift/burden [24]. We therefore further hypothesised that *life worthwhile* would correlate most highly with Spirit 8 items related to meaning and the value of life, i.e. (iv) Q6 (‘I have a better sense of meaning now than I have had in the past’) and (v) Q8 (‘Life has become more precious to me; every day is a gift’). As many items in the Spirit 8 relate to preparation for death rather than meaning, we also hypothesised that (vi) *peace* would correlate more strongly than *life worthwhile* with Spirit 8 *spiritual well*-*being*. However, owing to the different timescales and scoring formats of the POS and Spirit 8 items we hypothesised that correlations would be at best moderate. Finally, we hypothesised that (vii) *peace* and *life worthwhile* would be correlated at least moderately strongly owing to the results of the recent factor analysis, in which these items formed a single existential factor (loading at 0.59 and 0.90 respectively) in the three-factor model with best fit [28]. Following [41], a correlation of ≥0.5 was interpreted as strong, 0.5-0.3 as moderate, and 0.3-0.1 as weak. Moderate or strong correlations between items were taken as evidence of conceptual overlap in the constructs measured by the items. Significance was set at p < 0.01 to account for multiple testing.

Findings from the thematic content analysis and correlation analyses were integrated through triangulation [42], comparing and contrasting the findings to determine what *peace* and *life worthwhile* measure and how the items relate to each other.

## Results

### Patient characteristics

Characteristics of participants in cognitive interviews (n = 72) and quantitative data collection (n = 285) are presented in Table 1. About two thirds were women. Among cognitive interview participants, 60% had a primary diagnosis of HIV and 39% cancer; among survey participants 81% had HIV and 18% cancer. Cognitive interview participants were older (mean age 45 compared with 40). The majority of cognitive interview participants (n = 55) were of a Christian faith, with a wide range of church affiliations reported.

**Table 1:** Demographic characteristics

**Demographic characteristic**	**Cognitive interviews (N = 72)**	**Survey (N = 285)**
Age		
*Mean (SD)*	45.1 (15.9)	40.1 (12.8)
*Median*	42.0	38.0
*Range*	18-84	18-88
Gender		
*Women N (%)*	48 (66.7)	197 (69.1)
Primary diagnosis N (%)		
*HIV only*	43 (59.7)	230 (80.7)
*Cancer only*	28 (38.9)	51 (17.9)
*MND*	1 (1.4)	0
*Multiple sclerosis*	0	1 (0.4)
*Systemic lupus erythematosus*	0	1 (0.4)
*Korsakoff’s syndrome*	0	1 (0.4)
*Of HIV + patients:*		
On ART N (%)	24 (55.8)	127 (55.2)^a^
Prior AIDS diagnosis N (%)	37 (86.0)	192 (83.5)
Dual HIV-cancer diagnosis N (%)	5 (11.6)	35 (15.2)
ECOG Functional status N (%)		
*Fully active*	8 (11.1)	21 (7.4)
*Restricted*	24 (33.3)	65 (22.8)
*Ambulatory*	17 (23.6)	79 (27.7)
*Limited self care*	13 (18.1)	87 (30.5)
*Completely disabled*	10 (13.9)	33 (11.6)
Primary place of palliative care N (%)		
*Home*	49 (68.1)	180 (63.2)
*Inpatient*	16 (22.2)	74 (26)
*Outpatient*	3 (4.2)	13 (4.6)
*Day care*	4 (5.6)	18 (6.3)
Weeks under palliative care		
*Mean (SD)*	63.5 (91.6)^a^	46.0 (74.8)
*Median*	26.0	12.0
*Range*	1-427^a^	0-468
If Christian, church affiliation (where described) N (%)		Not available
*Catholic*	11 (20)	
*Anglican*	5 (9.1)	
*Pentecostal*	5 (9.1)	
*Methodist*	4 (7.3)	
*Dutch reformed church*	2 (3.6)	
*United Presbyterian-congregational*	1 (1.8)	
*Calvinist*	1 (1.8)	
*Christian, not stated*	24 (43.6)	
Other*	2 (3.6)	
Responsible for children?		
*Yes N (%)*	53 (73.6)	232 (81.4)
*If yes, mean no. children (SD)*	2.9 (1.8)	3.1 (2.0)
*If yes, median*	2.0	3.0
*If yes, range*	1-9	1-12
Household size		
*Mean (SD)*	4.9 (3.1)^a^	5.3 (2.5)^b^
*Median*	4.0	5.0
*Range*	1-20^a^	1-14^b^
Location of home N (%)		
*Urban*	23 (31.9)	53 (18.6)
*Peri-urban*	26 (36.1)	53 (18.6)
*Rural*	23 (31.9)	179 (62.8)

^a^ Missing =1

^b^ Missing =3

* Christian Mission Church and Universal Church

### Objective (1): content validity

Both *peace* and *life worthwhile* items were well understood by patients. Patients interpreted ‘feeling at peace’ according to four main themes (presented with exemplifying quotations in Table 2), often discussing the concept in relation to two or more of these interpretations.

**Table 2:** Patients’ interpretations of ’peace’ and ‘life worthwhile’

**Interpretations of ‘peace’ and exemplifying quotations**	**Interpretations of ‘life worthwhile’ and exemplifying quotations**
**Perception of self and world**	**Perception of self and world**
• Peace as a feeling (not worrying, being happy)	• Life worthwhile as a feeling (not feeling down, feeling happy)
I stay peacefully these days because I have no worries. This makes me go day by day. (A-HO030, Uganda)	They are trying to find out how positive we are. Whether we feel down all the time or whether we feel happy about life. (D007, South Africa)
• Peace as an attitude (acceptance, hope)	• Life worthwhile as an attitude (being positive, not giving up)
With this it’s a mental thing, I think, and it’s about acceptance… I think the whole thing with people and cancer is, ‘I’ve got cancer, what‘s going to happen to me, what the hell am I going to do?’ (D017, South Africa)	Life is worthwhile big time, because one must not say because I am sick I give up on life and lose hope… You must be strong in life and face facts, don’t be shy, and go for it. (C073, South Africa)
• Peace as an experience (relaxing, calming)	• Life worthwhile as the importance of life
When I’m on the beach, when I’m sitting on the rocks and the dog is running in and out of the water, that’s peace. (D004, South Africa)	*Interviewer: What do you think is the meaning of this question?*
	A-HO031: That has life been important to me? Yes, I feel that my life is very important. That’s why I have the courage to live up to this time otherwise with what I faced I would have gone long ago. (Uganda)
	• Life worthwhile as meaning, purpose
	*Interviewer: What do you think the question means?*
	A-KA029: (Laughs then goes quiet) Maybe it means does it have purpose. (Uganda)
	I think it means that was my life meaningful in the past three days. (C066, Christian woman with HIV, 37 years old, South Africa)
	• Life worthwhile as quality of life
	It depends on how thing are going and your quality of life I think… It depends on how the day is going really, and how I’m feeling. (D012, South Africa)
**Relationship to others**	**Relationship to others**
• Peace as harmonious, forgiving relationships	• Life worthwhile as value to others
Maybe they mean how do I feel I cope with the others, like my neighbours [community], is there peace within us? (D069, South Africa)	It means may be on my side do I still love my life? Or is it valuable to others? (A-KA030, Uganda) As I was there in hospital I dreamt of seeing this brother and my son. We were very, very happy together. I then realised that there are still people who love me. On waking up I then realised I don’t want to die. It was then my life took a turn for the positive. (B-LL033, South Africa)
• Peace as supportive relationships	
*Interviewer: What does it then mean to you to feel at peace?*	
A-KA023: This is because my relatives regularly check on me and they provide medication and food at least making me happy. They don’t leave me alone. (Uganda)	
• Peace as openness with others	• Life worthwhile as togetherness
*Interviewer: What sort of things would you think about to help you answer that?*	[It is asking] if you are happy with your family or your children and you want to spend more time with them. Something to look forward to everyday. Your children are married and your grandchildren are growing up and they love you so much, they come to you and they cling to you. You feel that you want to be with them forever. (D066, South Africa)
D007: Don’t carry it all on my own, try to share with others. Especially with my husband… which I’m not always able to do. Ja… If someone’s very healthy they don’t want to know about your moans and groans. (South Africa)	
**Spiritual beliefs**	**Identity**
• Peace as comfort from belief in afterlife	• Life worthwhile as independence
I know my God. I know, though still young, that one day I will go to eternal life. I am at peace and I know that I will be going to a better place to my husband. (B-LK012, South Africa)	*Interviewer: Do you feel that your life has been worthwhile? Do you understand the question?*
	B-LK014: Yes, I have been doing things for myself. I was never dependent on anybody. I want to get better and regain my strength and be able to work again. (South Africa)
• Peace as faith in God	• Life worthwhile as ability to work
Yes, I am very much at peace. I know God loves me. If not so I would not be feeling as I am. (B-LL032, South Africa)	Yes, my life was valuable to me. I was working, had a lot of plans for my future and was very well until I started being sick with TB. Since then I thought if I have TB something else is going to follow. It is worse when I heard that I am HIV positive. I am just living each day – nothing has value anymore. (B-HH011, South Africa)
I’m not religious, as I said before, but maybe make peace with your maker, that sort of thing. (D002, South Africa)	
• Peace as support from ancestors	
Yes, I am at peace. I have accepted everything and more because I know that my ancestors have not abandoned me. (D061, South Africa)	
**Health and healthcare**	
• Peace as pain and symptom control	
*Interviewer: Have you felt at peace – inner peace?*	
B-HH014: Yes, I am satisfied. I get my medication and that makes me feel good. (South Africa)	
How can I get peace when I am feeling pain? The peace I have is because of care and support from I got from the Hospice. (A-HO030, Uganda)	
• Peace as health	
I cannot say I am at total peace because I am still not well. My state of health is not where I want it to be but as I have said, I believe God is going to give me back my health and strength. (B-LL036, South Africa)	
• Peace as full information	
Yes, I did have peace, when they have explained about everything. (C065, South Africa)	

In the first theme, patients described feeling at peace in relation to their perception of self and the world. Peace was described as feeling relaxed, happy, positive and free, forgiving of others, and not worrying, grieving or feeling disturbed. Patients also discussed peace as an attitude, the need to forgive themselves and others and accept their life situation to reach a sense of peace and hope. However, this did not always entail accepting that their illness was incurable (D067). Peace as an experience was less frequently expressed by patients, but powerful images were described. One patient talked about experiencing peace alone in nature (D004). For a Hindi woman, the experience of peace related to listening to prayers and spending time with her grandson (D005).

In the second theme, peace as relationship to others, patients discussed harmonious, forgiving, supportive and open relationships with their friends, family and communities. Where patients reported problematic relationships with family members (for example, stigma), this affected their sense of peace (B-LL028). Patients who reported family support described how this helped them feel at peace (B-LL035, A-KA023) and how being accepted by others helped them accept themselves (A-KA025). One man in Uganda talked about how losing family members to AIDS had diminished his circle of support and prevented him from feeling at peace (A-HO031). Difficulties communicating with family members and friends were particularly acute when patients feared disclosing an HIV diagnosis (e.g. D041), but disclosure facilitated feeling at peace (C071).

In the third theme, spiritual beliefs, patients described peace in term of their ability to relate to God and the ancestors (B-LL037, D061) and the extent to which they felt supported by their beliefs, particularly in an afterlife. One patient interpreted the peace item to be about religion, although he did not see himself as religious (D002).

The fourth theme concerned interpretations of peace that related to health and healthcare (B-HH014, B-LL036, A-HO030), for example good pain and symptom management and receiving full information about the illness (C065).

Patients’ interpretations of ‘feeling life is worthwhile’ occurred in three main themes (Table 2). In the first theme, patients discussed feeling life worthwhile in terms of their feelings about life and attitude to their illness and its consequences. Related to this were interpretations of life worthwhile as the importance or meaningfulness of life and quality of life. Participants defined quality of life in terms of other factors that made life worthwhile (e.g. independence and their relationships with others). For example, one woman related life being worthwhile to her relationship with her son (D004).

The second theme, life worthwhile as relationship to others, referred to the way in which relationships with family members brought a sense of being valued, something to live for, and intimacy or togetherness. A woman in Cape Town who had experienced rape and other trauma found the question of whether she felt life was worthwhile difficult to answer, but described the importance of acceptance and the love of others in preventing a suicide attempt (B-LL033).

Patients interpreted life worthwhile in relation to the third theme, personal identity, when they described their sense of life worth as reliant on independence (both physical and financial) and their ability to work. Such descriptions were closely related to patients’ sense of usefulness and ability to find meaning in their lives. Ability to work represented financial independence, but also the capacity to live a full, valuable and meaningful life (e.g. D017).

### Objective (2): construct validity

Correlations of POS *life worthwhile* and *peace* with each other and Spirit 8 *spiritual well*-*being* and individual items are presented in Table 3. Hypotheses (i) and (iii-vii) were met, with *peace* correlating most strongly with Spirit 8 Q1 and Q7 and *life worthwhile* correlating most strongly with Q6 and Q8 (all r = 0.34, p < 0.001). *Spiritual well*-*being* was moderately correlated with *peace* (r = 0.46, p < 0.01) but only weakly with *life worthwhile* (r = 0.18, p < 0.001). Hypothesis (ii) was not met, with *peace* and Q2 correlating only weakly (r = 0.22, p < 0.01).

**Table 3:** Correlation matrix

	***Life worthwhile***	***Peace***
***Peace***	.35*	-
**Spirit 8 *spiritual well-being***	.18*	.46*
**Spirit 8 Q1**^**a **^**(My affairs are in order; I could die today with clear mind)**	.038	.34*
**Spirit 8 Q2**^**a **^**(I feel generally at peace and prepared to leave this life)**	-.05	.22*
**Spirit 8 Q3 (I am more satisfied with myself as a person now than I was before my illness)**	.04	.27*
**Spirit 8 Q4**^**a **^**(The longer I am ill, the more comfortable I am with the idea of ‘letting go’)**	.08	.23*
**Spirit 8 Q5**^**a **^**(I have a greater sense of connection to all things now than I did before my illness)**	.13	.18*
**Spirit 8 Q6**^**a **^**(I have a better sense of meaning in my life now than I have had in the past)**	.34*	.16*
**Spirit 8 Q7**^**a **^**(As end of life approaches, I am comfortable with the thought of my own death)**	-.03	.34*
**Spirit 8 Q8**^**a **^**(Life has become more precious to me; every day is a gift)**	.34*	.20*

*Correlation is significant at the 0.01 level.

Correlations where r > 0.30 (i.e. of at least moderate magnitude) are highlighted in bold.

^a^ Spirit 8 items involve choosing between positive and negative statements; only the positive statements are presented here.

The matrix shows correlations of Spirit 8 spiritual well-being and individual items with APCA African POS *peace* and *life worthwhile* (Spearman’s rho, N = 277 to N = 285).

**Figure 1:**
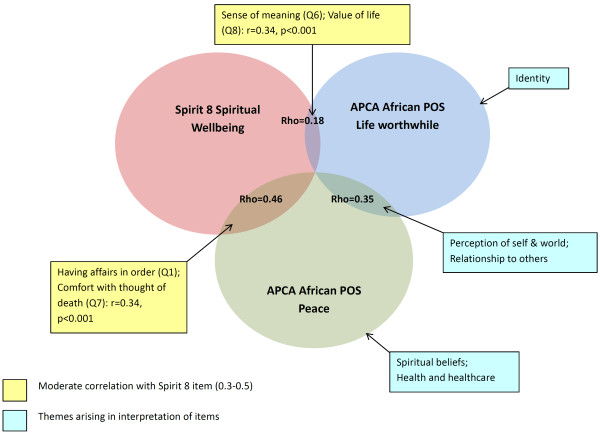
**Relationships between *peace *and *life worthwhile *and Spirit 8 *spiritual well*-*being*.**

Integrated findings from objectives (1) and (2) are modeled in Figure 1, which displays the Spirit 8 items most strongly correlated with *peace* and *life worthwhile* and interpretations of the POS items by theme.

## Discussion

This study provides, for the first time, an understanding of the meaning and constructs of ‘feeling at peace’ and ‘feeling life is worthwhile’ in sub-Saharan African advanced disease populations. The novel, patient-centred approach demonstrates the complex nature of these concepts and the value of using mixed methods to assess the validity of measurement tools. The findings demonstrate that these POS items measure related but different aspects of spiritual well-being, and suggest that these items can be used to identify spiritual need and measure spiritual outcomes in this population, which displays high levels of spiritual distress [16].

We found both similarities and differences in the constructs assessed by *peace* and *life worthwhile* and Spirit 8 *spiritual well*-*being* and individual items (Figure 1). Although correlation between *peace* and *life worthwhile* was only moderate (r = 0.35), the cognitive interview data highlight a degree of conceptual overlap which supports the loading of these items on an existential well-being factor in the recent factor analysis of the tool [28]. Both ‘feeling at peace’ and ‘feeling life is worthwhile’ were interpreted in terms of patients’ perception of life and the world and relationships to others. A recent study in Uganda found that hospice staff also had a relational understanding of patients’ spiritual well-being in terms of interconnectedness and a person’s relationship to their culture, society, neighbours and family [43]. The influence of social relationships on spiritual well-being reflects the collectivism of African culture and the principle of *Ubuntu*, which appears in many African languages and expresses the view that the essence of being human is interconnectedness and one cannot exist as a human being in isolation [44]. The collectivistic aspects of African culture may make stigma even more difficult to cope with than in more individualistic societies [45].

The findings also demonstrate the divergence between the constructs of *peace* and *life worthwhile*. There was moderate correlation between the Spirit 8 *spiritual well*-*being* score and *peace* (r = 0.46), but little correlation between *life worthwhile* and *spiritual well*-*being* (r = 0.18). Constituent items of the Spirit 8 refer to spiritual, psychological and relational preparation for death and dying, reflecting the development of the original MVQOLI as a tool to assess quality of life at the end of life rather than in a chronic disease HIV population [33]. *Peace* but not *life worthwhile* also appears to be related to preparation for death, with *peace* moderately correlated with having one’s affairs in order (Spirit 8 Q1) and to comfort/unease with the thought of one’s death (Q7) (both r = 0.34, p < 0.001). This is reflected in patients’ interpretations of peace as involving forgiveness and acceptance, which are fundamental to preparation for death [46], and drawing on spiritual beliefs. The concept of peace was also associated with being at peace with others in van der Geest’s study in Ghana [47].

However, it is interesting that *peace* was only weakly correlated with Spirit 8 Q2 (‘I feel generally at peace and prepared to leave this life’) (r = 0.22). This highlights two important points: first, ‘feeling at peace’ is interpreted in many different ways in this population, not all of which relate directly to preparation for death; for example, peace in the ‘here and now’ rather than in some future, hoped-for state of being after death. This may be due to HIV now being a chronic rather than terminal condition, but also other factors; for example, a cultural reticence to talk about death could make agreement with the phrase ‘prepared to leave this life’ problematic. Alternatively, this conceptualisation of feeling at peace could display a North American bias; indeed, the way Spirit 8 items 1, 2 and 7 conceptually combine how the patient currently feels with preparation for death might be problematic in this population. This warrants further exploration. Second, the specified timeframe may be important when asking someone whether they feel at peace. While the POS refers to the three days prior to assessment, the Spirit 8 refers to the present, and this time difference may have affected levels of correlation. The factors that patients think about when asked whether they feel at peace now may be very different from those they think about when considering the past three days. Asking about peace during a specified period could be perceived as a less existential and more down-to-earth question than asking about peace *per se*. This hypothesis is supported by the qualitative finding that patients also interpreted *peace* in terms of every-day concerns and experiences: socioeconomic worries, pain and other symptoms, and experiences of calm and togetherness. It is also supported by the results of the factor analysis of the POS, which found that peace loaded on the physical and psychological well-being factor (0.51) only slightly less than on the existential factor (0.59) [28].

In contrast, patients interpreted life being worthwhile in abstract terms, describing the meaning, value, purpose or quality of their lives. These interpretations support the significant moderate correlations between *life worthwhile* and Spirit 8 Q6 (having a better/worse sense of meaning in life compared with in the past) and Q8 (the value/burden of life) (both r = 0.34, p < 0.001). The relationship of life worthwhile to the perception of life as gift or burden is found in interpretations of *life worthwhile* in attitudinal terms (hope, gratitude). Patients’ appraisals of life as worthwhile were also related to identity: their ability to work, to be independent, valued by others and plan for the future, or, conversely, to feelings of helplessness or uselessness.

Steinhauser et al. explored the meaning and clinical usefulness of the question ‘Do you feel at peace?’ in patients with incurable, progressive disease in the USA. Echoing findings from this study, peace was perceived as a result of good clinical care and as a consequence of resolved conflicts with family members, within themselves, in their relationship with God, or in spiritual reflection on the meaning of illness [48]. Steinhauser et al. also found that items measuring peacefulness correlated highly with having a chance to say goodbye, making a positive difference in the lives of others, giving to others, sharing one’s deepest thoughts, and having a sense of meaning [49]. Peacefulness as measured by the QUAL-E (‘I feel at peace’) had moderate to strong correlations with the tool’s emotional, spiritual and social well-being subscales and with purpose and faith dimensions of the FACIT-Sp [40]. These associations were evident in our study, in which peace related to interpersonal and social factors, feeling life is worthwhile, preparation for death, and faith.

### Limitations

The mixed-methods study design and the large sample size are assets of the study, but there are limitations relating to translation and sampling. The MVQOLI (and hence the Spirit 8 derived from it) was originally developed in the USA and re-validated in Uganda [34]. Owing to resource constraints, we were unable to use the best practice methods of tool adaptation: synthesis of multiple translations, back translation, expert review and pretesting prior to psychometric testing [50]. However, we minimised misinterpretations by using academic departments, staff at the participating services, and independent peer review. Similarly, while one of the strengths of the study is that the cognitive interview data were collected, transcribed and translated by local researchers with cultural insight, the translated interview transcripts were not back-translated [51]. Instead, all translations were cross-checked by bilingual palliative care staff and difficulties in translation resolved through discussion with the local research team.

The sampling frame in the cognitive interview phase generated a maximum variation sample, while consecutive sampling was used in the quantitative phase. This resulted in a higher proportion of cancer patients in the cognitive interview sample, which also represented an equal spread of patients from rural, urban and peri-urban areas, while quantitative data were predominantly from rural areas. One reason for this is the inclusion in the quantitative data collection of an additional rural facility serving HIV patients that did not participate in the cognitive interviews. However, another facility serving HIV patients in the same province was included in the cognitive interviewing, so this perspective was not omitted. The use of consecutive rather than random sampling in the survey phase was owing to resource and population restrictions and may have a potential sampling bias. In addition, although we were able to recruit a diverse sample with respect to languages and care settings, the use of uniform self-report data collection in the survey (to minimise measurement bias introduced by using both staff and patient rating) may have introduced a sampling bias in that those with very poor health status could not participate. Finally, the sample was not sufficiently large to examine variations in responses due to cultural and linguistic differences; further research is needed in this area.

### Implications for clinical practice and research

Findings demonstrate that *peace* and *life worthwhile* are distinct but related items that elicit holistic concerns and are not prescriptive (i.e. do not embody a world view biased to a particular religious outlook). As the items are also brief, easy to administer and are comprehended well by patients, it is recommended that they are used in routine assessment to focus the attention of the care team on what really matters to the patient and screen for spiritual distress. Use of the items may thus help ensure that care is patient-centred and equitable and that needs for spiritual support are not conflated with religious needs. Patients scoring poorly on either or both of the items (following the procedure adopted for depression [52]) could be immediately assessed in a more in-depth way and referred as necessary. A similar approach using an ‘at peace’ item to initiate discussion of holistic concerns has been suggested by Steinhauser et al. [40]. Using broad questions such as *peace* and/or *life worthwhile* is advantageous as clinicians can adapt their subsequent conversation and response according to the patient’s own interpretation of the question. This could also have application for countries outside of sub-Saharan Africa. Palliative care providers in sub-Saharan Africa are referred to recent recommendations regarding spiritual care and assessment [53].

### Future research

The effectiveness of using the POS items to screen for spiritual distress warrants further investigation in studies examining the cut-off scores which indicate need for spiritual support. Further research is also required to determine the acceptability to staff and patients of incorporating POS items into patient assessment and a referral pathway for responding to identified needs. The POS is currently being used in clinical practice and research studies across Africa, hence there is scope for future large-scale multicentre studies of this nature. While this study has confirmed that *peace* and *life worthwhile* measure aspects of spiritual well-being, fully establishing content validity would require research investigating the extent to which *peace* and *life worthwhile* are comprehensive, i.e. capture all aspects of spiritual well-being in this population and in different cultural and linguistic groups. Finally, the similarities regarding interpretations of peace in the USA and in South Africa and Uganda point to the possibility that peace is a cross-culturally applicable concept which may be useful in assessing and comparing patients’ holistic well-being and needs across diverse cultural contexts, and this warrants further research.

## Conclusion

The APCA African POS items *peace* and *life worthwhile* capture distinct but related constructs which indicate the extent of a patient’s spiritual well-being in palliative care populations in sub-Saharan Africa. The items can be used to elicit holistic concerns including spiritual distress, and are brief and simple enough to be integrated into routine practice.

## Competing interests

The authors declare that they have no competing interests.

## Authors’ contributions

LS designed and managed the study, analysed the data and drafted the manuscript. PS contributed to data analysis. MG and JD participated in the design of the study. GA, TM and KM collected the data. LG, TM, LMS and BI oversaw and supported local data collection. RH and IJH contributed to study design and obtained funding. All authors read and approved the final manuscript.
